# Evaluating nurse plants for restoring native woody species to degraded subtropical woodlands

**DOI:** 10.1002/ece3.1294

**Published:** 2014-12-23

**Authors:** Stephanie G Yelenik, Nicole DiManno, Carla M D'Antonio

**Affiliations:** 1 U.S. Geological Survey, Pacific Island Ecosystems Research CenterHawai'i National Park, Hawai'i; 2 Hawai'i Cooperative Studies Unit, University of Hawai'iHilo, Hawai'i; 3 Environmental Studies, University of CaliforniaSanta Barbara, California

**Keywords:** competition, *Dodonaea viscosa*, dry subtropical *Metrosideros* woodland, facilitation, invasive grasses, *Leptocophylla tameiaeia*, *Morella faya*, restoration, seedling recruitment, succession

## Abstract

Harsh habitats dominated by invasive species are difficult to restore. Invasive grasses in arid environments slow succession toward more desired composition, yet grass removal exacerbates high light and temperature, making the use of “nurse plants” an appealing strategy. In this study of degraded subtropical woodlands dominated by alien grasses in Hawai'i, we evaluated whether individuals of two native (*Dodonaea viscosa*, *Leptocophylla tameiameia*) and one non-native (*Morella faya*) woody species (1) act as natural nodes of recruitment for native woody species and (2) can be used to enhance survivorship of outplanted native woody species. To address these questions, we quantified the presence and persistence of seedlings naturally recruiting beneath adult nurse shrubs and compared survival and growth of experimentally outplanted seedlings of seven native woody species under the nurse species compared to intact and cleared alien-grass plots. We found that the two native nurse shrubs recruit their own offspring, but do not act as establishment nodes for other species. *Morella faya* recruited even fewer seedlings than native shrubs. Thus, outplanting will be necessary to increase abundance and diversity of native woody species. Outplant survival was the highest under shrubs compared to away from them with few differences between nurse species. The worst habitat for native seedling survival and growth was within the unmanaged invasive grass matrix. Although the two native nurse species did not differentially affect outplant survival, *D. viscosa* is the most widespread and easily propagated and is thus more likely to be useful as an initial nurse species. The outplanted species showed variable responses to nurse habitats that we attribute to resource requirements resulting from their typical successional stage and nitrogen fixation capability.

## Introduction

Numerous studies have documented priority effects whereby established plants suppress newly establishing individuals (e.g., D'Antonio et al. 2001; Corbin and D'Antonio 2004). Yet, these same incumbents can have positive effects on establishing species by increasing shade, nutrients, and protection from herbivores (Chapin et al. 1994; Callaway and Walker 1997). It is now well accepted that competitive and facilitative processes change in strength and direction across environmental gradients, over time, and across species pairs, making net outcomes difficult to predict (Miriti 2006; Maestre et al. 2009). By understanding contingencies influencing these opposing interactions, ecologists can better predict species interactions, potentially to the benefit of restoration efforts (Padilla and Pugnaire 2006; Brooker et al. 2007).

Restoration practitioners are increasingly interested in using pre-established plants to facilitate the success of individuals outplanted into degraded, harsh habitats (Padilla and Pugnaire 2006). Planting focal species under nurse species can increase survivorship and growth if facilitative effects outweigh competitive ones (Gomez-Aparicio et al. 2004; Padilla and Pugnaire 2006). A facilitative nurse effect can result from aboveground processes including shading from harsh sun, protection from wind, temperature extremes and herbivores, lowered water stress, and increased pollinator visitation (Yang et al. 2010; Smit and Ruifrok 2011). Belowground effects include increased water and nutrient availability, reduced soil compaction/erosion, and increased microorganism activity (Yang et al. 2010; Smit and Ruifrok 2011).

The strength and balance of competitive and facilitative processes are affected by the identity and traits of the nurse species. In turn, the identity of the target outplant species can be important in determining the net effect because species vary in their resource requirements and have different sensitivities to abiotic stressors and pathogens (Gomez-Aparicio et al. 2004). For example, incumbent nitrogen (N) fixing trees or shrubs are likely to increase soil N, which would be an important facilitative process in an N-limited ecosystem (Gomez-Aparicio et al. 2004), but to take advantage of this, a desired outplant species would need to tolerate the shade, litter, and potential allelochemicals produced by the fixer. A recent meta-analysis of nurse plants and their role in restoration (Gomez-Aparicio 2009) provided support for Connell and Slatyer's (1977) model of succession by facilitation in which late-successional target species, predominantly shrubs and trees, benefited the most from nurse plants because of their shade tolerance and their generally good competitive abilities.

In addition to being useful as outplanting sites, established plants may also provide places where wind-blown seed accumulates, refugia for naturally recruiting seedlings, and sources of seed for the colonization of surrounding areas. Established plants could thus become nurse plants for native woody species, and therefore nodes for community development (Corbin and Holl 2012). Understanding whether nurse plants play a role in natural regeneration is helpful for predicting which plant assemblages are likely to form around incumbents. However, few studies match outplant studies under nurse plants with natural recruitment patterns, even though such combined information would not only inform decisions about active restoration strategies, but also predict longer-term community trajectories.

Dry tropical and subtropical forests/woodlands are among the most degraded habitats worldwide with large areas converted to pasture, grazed savanna, or other human uses (e.g., Murphy and Lugo 1986; Pennington et al. 2006). Although these ecosystems are notoriously difficult to restore due to harsh conditions, low seedling survival rates, and depauperate seed banks, there are few studies that document the use of nurse plants in such settings (although see Aerts et al. 2007; Medeiros et al. 2014). In Hawai'i, dry forests/woodlands have been reduced to a much greater extent than wet forests (Pratt and Gon 1998) and there is a paucity of knowledge about how to successfully restore them (Cabin et al. 2002b; Vieira and Scariot 2006). Here, we evaluate the potential for using established woody plants as nurse plants in Hawai'i for restoring desired species to dry *Metrosideros* woodlands degraded by invasive grasses and fire. Our results are broadly applicable because the strong seasonality of rainfall and high temperatures characterizing the region (Giambelluca et al. 2013) and the abundance of exotic grasses are typical for degraded subtropical and tropical savannas and woodlands including ones in Australia (Lonsdale 1994; Rossiter et al. 2003), Central America (Janzen 1988), and Brazil (Klink and Machado 2005). Such conditions create a challenging environment for establishment of woody species, particularly later successional ones.

The objective of this study was to evaluate whether the woody species present on this degraded landscape*—Dodonaea viscosa* (Jacq.)*, Leptocophylla tameiameia* (Cham. & Schltdl.), and *Morella faya* (Ait.)—could serve as nurse plants for the purpose of establishing desired native shrubs and trees. Our study is unique in that the former two species are native, but the latter is from the Azores and is highly invasive. Its common occurrence in the region, and ability to fix N, however, spurred our interest in whether it could facilitate other species, temporarily, prior to being removed. If so, then exotic species removal could be combined with outplanting. Specifically, we asked: (1) Do different established adult species increase natural regeneration of native shrub/tree seedlings in their vicinity? (2) To what degree do different potential nurse plants increase survivorship of outplanted seedlings of desired species? and (3) What characteristics make established plants good facilitators? To answer these questions, we: (a) evaluated both natural regeneration and experimental outplant survival under the three potential nurse species compared to the existing exotic grass, *Melinis minutiflora*, and to areas cleared of grass as might happen in an intensive restoration effort, (b) characterized soil and canopy traits of potential nurse species compared to open and grass-covered sites, and (c) evaluated growth of target seedlings in potential nurse plant soil in a controlled setting. Throughout the manuscript, the term “nurse plant species” refers to adults of *D. viscosa*, *L. tameiameia*, *and M. faya*, as opposed to the exotic grass habitats (hereafter, “*Melinis* matrix”).

## Methods

### Study sites and species

**Figure 1:**
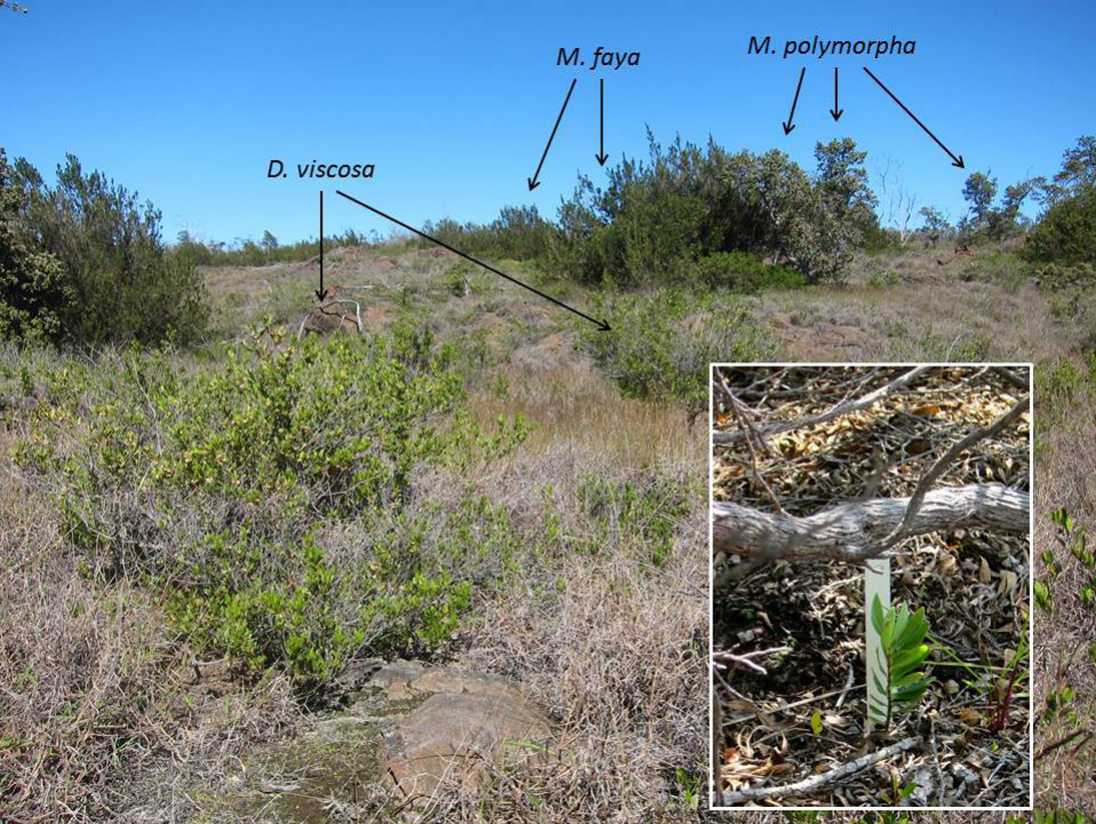
Photograph of degraded *Metrosideros polymorpha* woodland showing native *Dodonaea viscosa* shrubs and invasive *Melinis minutiflora* grass matrix in the foreground, and *M. polymorpha* (new foliage has light green tips) and invasive *Morella faya* (dark green foliage only) in the background. Inset shows an outplanted *Wikstroemia phillyreifolia* seedling approximately 8 cm tall under a *D. viscosa* nurse shrub after 2 years. Main photograph: S. Yelenik, inset photograph: C. D'Antonio.

Study sites were located in Hawai′i Volcanoes National Park on Hawai′i Island (19°6′N, 155°33′W, ∼900 m elevation). The climate is seasonal, and the vegetation is classified as subtropical lower montane dry forest (http://www.fs.fed.us/psw/topics/ecosystem_processes/tropical/restoration/lifezone/hawaii/). Mean annual precipitation is 1.5 m, with most rainfall occurring between October and April, and mean temperatures at 22°C and 19°C for the summer and winter months, respectively. The surface soil is an entisol, derived from volcanic ash eruptions over the past 400 years. It overlies fine cinder and Pahoehoe lava bedrock that is between 750 and 1000 years old. The primary woodland prior to fire was dominated by the native canopy tree, *Metrosideros polymorpha* (Gaudich.), and an understory of native shrubs including all of our experimentally outplanted species except *Acacia koa* (A. Gray). Sites are described in detail elsewhere (Hughes et al. 1991; D'Antonio et al. 1998). Initial invasion by the exotic grass *Schizachyrium condensatum* (Kunth) increased fine fuel loads, leading to wildfires in 1970 and 1987. These fires resulted in the present community dominated by *M. minutiflora*, *D. viscosa*, and *M. faya* (Fig.1) (D'Antonio et al. 2011), although a small amount of *S. condensatum* (<1% cover) is still present. It should be noted that due to its windward location, young age of substrate, invasion, and wildfire history, this forest does not match descriptions for other dry forests in Hawai'i, which tend to have a higher diversity of canopy tree species rather than predominately *M. polymorpha*. Therefore, we refer to it as *M. polymorpha* woodland, although we also note that the abiotic conditions that lead to difficulty of restoration are similar to other dry forests in Hawai'i and elsewhere, making our results applicable to other ecosystems worldwide.

*Dodonaea viscosa* (Sapindaceae), the most common shrub, casts sparse shade and produces abundant small seeds within winged capsules. *Leptocophylla tameiameia* (Ericaceae), a less common shrub, forms a denser canopy and has fleshy fruits. *Morella faya* (Myricaceae), an exotic N-fixing tree, has invaded many areas of Hawai'i Volcanoes National Park. Individual trees grow to 7 m tall, form a dense canopy, and produce up to 20,000 seeds/tree/yr that are bird-dispersed (Vitousek and Walker 1989). We contrast these potential nurse species with swaths of the dominant African grass *Melinis minutiflor*a. This is a perennial, mat-forming species that reduces recruitment and growth of native shrub species (Hughes and Vitousek 1993; Yelenik and D'Antonio 2013). Hence, we do not expect it to be an effective nurse species, but it serves as the “control” matrix for regeneration conditions with no active restoration. Individuals are extremely difficult to distinguish; thus, we characterize the *Melinis* matrix, not individual plants.

### Experimental design and measurements

#### Nurse plant characterization and seedling recruitment

We characterized nurse plants and measured natural seedling recruitment in five vegetated swales (sites) dominated by *Melinis*, *D. viscosa*, and scattered *M. faya*. The sites were arbitrarily chosen, but separated from each other by protruding Pahoehoe lava and were at least 100 m apart. We characterized potential nurse shrubs using five replicates of each species (*D. viscosa*, *L. tameiameia,* and *M. faya*) per site, for a total number of 25 shrubs per species. We controlled for soil depth (at least 30 cm deep), but purposefully chose shrubs of varying sizes (within each species) in order to represent the common size range on the landscape (Table1). We placed two quadrats beneath the canopy of each shrub that were either 0.3 × 0.3 m or 0.5 × 0.5 m depending on shrub canopy size. We located one quadrat in low and one in high cover of *Melinis* to account for variability under shrub canopies. In the *Melinis* matrix between shrubs, we counted woody plant seedlings by establishing two 4 × 1 m plots at each of the five sites. We also added two additional *Melinis* matrix sites (*n* = 7) to account for low shrub recruitment in *Melinis* matrix.

**Table 1:** ANOVA statistics and means for abiotic characteristics of cleared *Melinis*, intact *Melinis*, *Dodonaea viscosa*, *Leptocophylla tameiameia*, and *Morella faya* habitats

	*F*	*P* *	Cleared	*Melinis*	*D. viscosa*	*L. tameiameia*	*M. faya*
Shrub variables							
Height (cm)	**369.3**_**(3,18)**_	**<0.001**	NA^1^	15.25_C_	115.88_B_	102.36_B_	360.08_A_
Width 1 (cm)	**67.1**_**(2,12)**_	**<0.001**	NA	NA	167.04_B_	158.12_B_	400.60_A_
Width 2 (cm)	**98.9**_**(2,12)**_	**<0.001**	NA	NA	142.88_B_	137.44_B_	424.00_A_
BD (cm)	**40.5**_**(2,12)**_	**<0.001**	NA	NA	4.34_B_	6.62_B_	23.77_A_
Volume (m^3^)	**61.6**_**(2,12)**_	**<0.001**	NA	NA	18.90_B_	10.92_B_	381.68_A_
Biomass (g)	**67.0**_**(2,12)**_	**<0.001**	NA	NA	1.37_B_	0.25_B_	11.97_A_
Litter depth (cm)	**4.8**_**(2,12)**_	**0.03**	NA	NA	3.64_AB_	2.40_B_	3.80_A_
Light (% transmittance)	**46.6**_**(4,30)**_	**<0.001**	97.2_A_	55.62_B_	35.15_c_	36.92_c_	28.05_c_
Soil variables							
Depth (cm)	0.7_(2,12)_	0.53	NA	NA	32.80	27.24	32.80
Temperature (°C)	**2.6**_**(4,30)**_	**0.06**	22.2_A_	20.68_AB_	20.11_B_	20.71_AB_	20.00_B_
Moisture (%)	0.9_(4,30)_	0.49	21.60	26.32	25.18	23.97	25.43
WHC (%)	1.7_(4,10)_	0.23	46.05	47.88	46.57	46.07	46.88
Organic matter (%)	**2.7**_**(4,29)**_	**0.05**	15.23_C_	20.40_ABC_	20.84_AB_	15.72_BC_	21.16_A_
Carbon (%)	**3.6**_**(4,30)**_	**0.02**	6.74_AB_	7.04_AB_	7.51_AB_	5.07_B_	11.71_A_
Nitrogen (%)	**3.3**_**(4,30)**_	**0.02**	0.37_AB_	0.38_AB_	0.38_AB_	0.33_B_	0.61_A_
C:N	**7.8**_**(4,30)**_	**<0.001**	17.6_AB_	18.38_A_	19.75_A_	15.22_B_	18.86_A_
Resin N Jan (ppm)	**2.4**_**(4,30)**_	**0.07**	5.57_B_	5.47_B_	5.36_B_	6.93_AB_	7.95_A_
Resin N March (ppm)	1.3_(4,30)_	0.29	6.54	9.19	8.38	10.11	9.04

* ANOVAs that resulted in *P* values <0.05 are bolded, and the corresponding letter designations reflect a Tukeys test. ANOVAs that resulted in *P* values <0.1 are also bolded, and the corresponding letter designations reflect a Student's T-test.

1 NA: Variables not measured.

We counted and marked all woody seedlings found in March 2012 under nurse shrubs or *Melinis* matrix. We identified seedlings to species and recorded the life stage. Life stages were classified as either first year (i.e., seedlings with cotyledons only or cotyledons and one set of true leaves) or 1+ years old (i.e., seedlings with true leaves only). It is possible that individuals with only true leaves may still be under 1 year old. In the *Melinis* matrix plots, we noted one of three categorical values for *Melinis* cover: open (∼0–20%), partial (∼20–80%), or full cover (∼80–100%). Each quadrat was revisited toward the end of the harshest months (September 2012) to estimate seedling survivorship and growth.

To further characterize nurse shrubs, we measured basal diameter, height, and canopy width and calculated biomass using allometric equations (Yelenik and D'Antonio 2013). We also visually quantified the percent cover of live and dead *Melinis*, bare rock, and vegetative debris (litter). Percent light interception was measured under shrub canopies using a Decagon AccuPAR model LP-80, soil and litter depth were measured using a soil probe, and soil temperature was measured in March 2012 using a VWR digital soil thermometer inserted into the top 10 cm.

#### Planting restoration target species under nurse plants

We propagated the following native species, ranging from common to threatened, for outplanting: *Acacia koa*, *Alphitonia ponderosa* (Hillebr.), *Dodonaea viscosa*, *Osteomeles anthyllidifolia* (Sm.), *Pittosporum terminaliodes* (Planch. ex Gray), *Sophora chrysophylla* (Salisb.), and *Wikstroemia phillyreifolia* (A. Gray) (see Tables S1 and S2 in Supporting Information). Seeds of all species were collected from plants growing as close to the study sites as possible with the exception of *A. koa*, which were collected from natural populations 400 m higher in elevation. Supplemental seeds (locally collected) of *S. chrysophylla* and *O. anthyllidifolia* were provided by the Park's Resources Management Division.

We imposed seed pretreatment following Lilleeng-Rosenberger (2005) and sowed seed in a perlite/vermiculite mix in summer 2011 in an outdoor greenhouse in the Park. When seedlings had two true leaves, we transplanted them to cone-tainers (164 mL volume) using a 3:1 mix of cinder: soil that had been sterilized and then inoculated with field-site soil. Seedlings were outplanted in December 2011 to the field sites.

We chose potential nurse plants at the seven sites used to measure natural recruitment in the *Melinis* matrix using the following criteria: standing alone (i.e., not close to other plants), soil 20 cm or deeper, and canopy large enough to cover all outplanted seedlings. We clipped *Melinis* growing under each nurse canopy and planted three individuals of *W. phillyreifolia* and *P. terminaliodes*, two of *A. ponderosa* and six of all others. We also planted seedlings into two *Melinis* treatments: intact *Melinis* and patches that were cleared by clipping stems at the soil surface. *Melinis* treatments also had soils that were 20 cm or deeper. We watered seedlings twice a week for 2 weeks during which we replaced any that died. Initial height and width measurements were recorded in January 2012, and survivorship was censused for 2 years (March, May, and September 2012; February 2013; and January 2014). Relative growth rates (RGR) were based on differences in seedling size between January 2012 and May 2012 and were calculated by modeling seedlings as the volume of an inverted cone. High mortality over summer 2012 restricted our ability to calculate meaningful longer-term RGR.

We measured the following characteristics of each nurse plant or *Melinis* treatment: light transmittance, soil and litter depth, soil temperature, soil moisture, soil water holding capacity (WHC), soil organic matter, soil carbon and nitrogen, and resin N availability. We collected soils using a 14.5 × 5 cm diameter core in February 2013 and sieved to 2 mm. We measured soil moisture gravimetrically by oven-drying at 100°C for 48 h, WHC by weighing the amount of water held in soils after being saturated in the laboratory and left to stand for 12 h, and quantified organic matter by combustion in a muffle furnace (500°C for 5 h). Soil samples for C and N were dried at 60°C, ground, and analyzed with a Costech CE Elemental Analyzer (Costech ECS 4010; Valencia, CA). We measured N availability with resin bags deployed in the field for 1 month periods in January 2012 and March 2012. After retrieval, the bags were extracted with 2 mol/L KCl (see DiStefano and Gholz 1986) and analyzed for 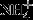 and 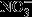 on a Lachat flow injection auto-analyzer. We only measured soil moisture at one time point; however, by choosing a winter date, we were confident that water would be available in this semi-arid region, allowing us to discern moisture differences due to species uptake, shading, or effects on soil properties. We attempted to measure soil moisture with a TDR on two other time points (spring, summer), but moisture values were too low (3%) in all sites for the TDR to give reliable values.

#### Greenhouse experiment

We collected soil to 10 cm from the outplant habitats to grow a subset of target seedlings away from the influence of a canopy. Soil was homogenized per “treatment” (*M. faya* soil (*n* = 8); *D. viscosa* soil (*n* = 10), *L. tameiameia* soil (*n* = 9); *Melinis* soil (*n* = 9)), sieved through 4-mm mesh and added to 1000 cm^3^ pots, and one seedling of *D. viscosa*, *W. phillyreifolia*, *S. chyrsophylla*, *or O. anthyllidifolia* was planted per pot. Other species were not included due to lack of seedling availability. Replication varied depending on soil availability. Seedlings were grown for 3 months, harvested, and weighed. Initial biomass was calculated from initial height using regression equations specific for each species, based on the final destructive harvest and height, in order to calculate relative growth rates (RGR). To compare N availability across soils, resin bags were placed in pots without seedlings (*n* = 4 per soil), collected after 1 month, and extracted with 2 mol/L KCl.

### Data analysis

#### Nurse plant characterization

JMP 9 (Cary, North Carolina, USA) was used for all statistical analyses. One-way ANOVA was used to analyze nurse plant characteristics in the natural recruitment study. Litter and soil depth data were compared using data from the recruitment study (*n* = 5). Light availability and the soil characteristics were measured at the habitats from the outplant experiment using means calculated per site (*n* = 7). Post hoc Tukey's tests were used to differentiate between different nurse species.

#### Natural recruitment patterns

To evaluate nurse habitat (*D. viscosa*, *L. tameiameia*, *M. faya*, *Melinis*) differences in seedling recruitment, we used a 1-way ANOVA with habitat as a fixed effect. Data were log-transformed for normality. We also used a 2-way ANOVA with nurse habitat and seedling species as fixed effects to assess whether recruiting seedling species tended to be associated with particular habitats. Similarly, we used a 2-way ANOVA with nurse habitat and seedling age as fixed effects to assess whether there were seedling life stage differences under various nurse shrub species. Finally, we tested how survivorship of seedlings differed as a function of nurse habitat using separate generalized linear models (glm) with a Poisson distribution for each life stage with nurse habitat and site as effects. We used Tukey's tests for post hoc analyses of ANOVAs and contrasts with sequential Bonferroni's adjustments for glms for all analyses in the study.

We used glms (Poisson distribution) to explore how abiotic variables that differed due to nurse shrubs affected first year seedling abundance and seedling survival. We added variables to glms in a forward stepwise fashion and chose the model with the lowest AIC score, and all significant variables. We used the variables: shrub biomass, *Melinis* percent cover, soil depth, litter depth, light transmittance, and soil temperature. Correlation analysis showed that none of the variables were correlated with an *r*^2^ ≥ 0.30, although some had slopes different than zero and we cannot rule out that variables were somewhat autocorrelated. Different variables were measured for the *Melinis* matrix: *Melinis* % cover, *Melinis* height, light transmittance, and soil temperature. *Melinis* percent cover and light were correlated (*r*^2^ = 0.38, *P* < 0.0001), so we used percent cover in the correlation analysis. The above variables were all taken on a per quadrat basis; however, in the first census, we also ranked the *Melinis* cover around each individual seedling as open, partial, or full. We then calculated proportion of seedlings in each site in each *Melinis* cover category and used glms (Poisson distribution) to assess differences in seedling occurrence between these *Melinis* categories. There were not enough surviving seedlings in the *Melinis* matrix to assess the importance of abiotic variables to seedling survival.

#### Outplant experiment

We used Survivorship Analysis of the 6 census dates to ask how nurse habitats affected survivorship of seedlings. We used post hoc contrasts with sequential Bonferroni's adjustments to assess differences between nurse habitats. To ask how different nurse habitats affected survivorship to 1 year and 2 years, or over the first summer of growth, we used glms (Poisson distribution). We used one-way ANOVAs to evaluate how nurse habitat affects seedling relative growth rates.

To evaluate how abiotic variables in our plots affected seedling outplant survivorship, we used glms (Poisson distribution) as described above. We included the variables: light transmittance, soil temperature, percent moisture, soil C:N, and January resin available N. Soil organic matter was not included because it was correlated (*r*^2^ = 0.37, *P* < 0.0001) with soil C:N. None of the other variables were correlated with an *r*^2^ > 3.0.

#### Greenhouse experiment

We used separate 1-way ANOVAs for each seedling species with soil origin as a fixed effect to evaluate how soils from different nurse environments affected seedling growth.

## Results

### Nurse plant characterization

The two native nurse species tended to be very similar to one another in all elements of their size (Table1). By contrast, *M. faya* was larger than the other nurse species, having greater height, width, basal diameter, and biomass (Table1). It tended to have lower light transmittance to the soil surface than the other species and had a deeper litter layer than *L. tameiameia*. The percent soil organic matter under *M. faya* was greater than that under *L. tameiameia* or where the *Melinis* matrix was cleared. Similarly, *M. faya* soil tended to have more C and N than all other soils particularly compared to *L. tameiameia*. Soil C:N was lowest under *L. tameiameia*.

Resin available N in January was greatest under *M. faya*, followed by *L. tameiameia*. By contrast, resin N measured in March did not differ between species. Soil temperature was lower under *M. faya* and *D. viscosa* than open habitats, although soil moisture and water holding capacity did not differ across the five habitats.

### Natural recruitment patterns

**Figure 2:**
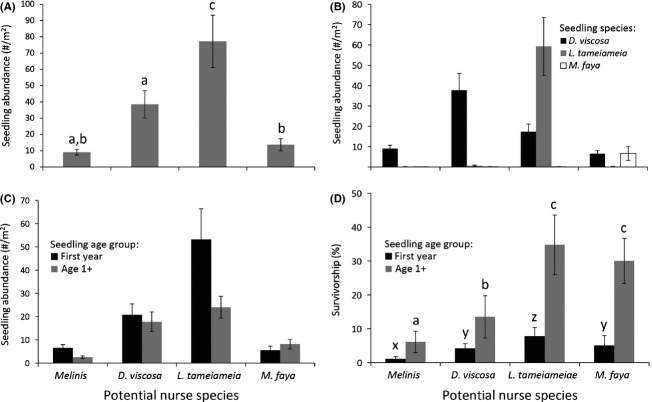
(A) Density of naturally recruiting seedlings under different nurse species. Seedling density under different nurse species as separated by (B) seedling species (*n* = 5, nurse habitat effect: *P* < 0.001, seedling species effect: *P* < 0.001, interaction: *P* < 0.001) and (C) seedling age (*n* = 5, nurse habitat effect: *P* < 0.001, seedling age effect: *P* = 0.03, interaction: *P* = 0.02). (D) Survivorship of naturally recruiting seedlings between 2011 and 2012 as a function of age class. Bars are means ± 1SE. Letters represent *P* < 0.05 for post hoc analyses in (A) and (D).

The largest number of seedlings occurred under *L. tameiameia* adults (Fig.2A), including recently germinated, and older cohorts (Fig.2C). Survival of marked seedlings was also the highest under *L. tameiameia* (Fig.2D). In contrast, *M. faya* adults had the fewest seedlings under their canopies, although this nurse species had the greatest diversity of seedling species, and relatively high survivorship (Fig.2A, D). The *Melinis* matrix had low numbers of seedlings and the lowest seedling survivorship of all habitats. Using categorical ranking of the *Melinis* environment (open, partial cover, full cover) around individual shrub seedlings, we found a greater percentage of seedlings under partial cover of *Melinis* (51% ± 6) than open (30% ± 8) or full cover (19% ± 3) microsites (*χ*^2^ = 77.39, *P* < 0.001).

Most seedlings were found under adults of the same species (Fig.2B), and these were found in dense populations. *Leptecophylla tameiameia* adults had, on average, 60 seedlings/m^2^ under their canopies, while *D. viscosa* had 38 seedlings/m^2^. Because seedlings were generally found only under adults of the same species, we could not disentangle nurse plant effects from species-specific seedling survival. One exception was for *D. viscosa* seedlings, which were found in all surveyed habitats, potentially due to their winged seeds. *Dodonaea viscosa* seedlings had higher survival under *L. tameiameia* (33%) and *M. faya* (31%) than under *D. viscosa* (10%) and within *Melinis* (2%) (1-way ANOVA with site as a random effect: *F*_3,14_ = 13.05, *P* < 0.001). Across species, first year seedlings had low survival across all microhabitats (4%), while older seedlings averaged 22% survival rates.

Different abiotic variables were important under the different nurse species (Table2A). *Melinis* cover was an important negative correlate of seedling abundance under *M. faya* and in the *Melinis* matrix, but was not important under *D. viscosa* or *L. tameiameia* despite its presence there. Results from the different nurse shrub species should be compared with caution because the seedling species under these shrubs varied. However, comparing between seedling abundance and survival provides clues to factors limiting later life stages. The positive or negative relationship between abiotic variables and seedlings sometimes switched as seedlings grew (Table2A, B). For example, a shift in sign was seen for the effect of soil depth on seedlings under *Dodonaea*: seedling abundance decreased with increasing soil depth while survivorship increased. Likewise, nurse shrub biomass, which was negatively correlated with light transmittance (*P* < 0.001, data not shown), was negatively associated with initial numbers of seedlings under *Dodonaea*, yet seedling survival was greater under bigger shrubs (Table2).

**Table 2:** Final glm models explaining variability in natural seedling abundance and survivorship under different nurse plant species

Nurse shrub species	Potential Model Effects*						AIC
	Shrub biomass (g)	*Melinis* cover (%)	Soil depth (cm)	Litter depth (cm)	Light (%)	Soil Temp (%)	
(A) First year seedling abundance							
*Dodonaea*	−0.0003		−0.0124	−0.2010	−0.0160		1881.16
*Leptocophylla*	+0.0034		−0.0280	−0.0622	+0.0217	+0.1708	3851.97
*Morella*	+0.0002	−0.0054	−0.0097	−0.1628			784.19
*Melinis*	NA^1^	−0.0363	NA	NA	NA	+0.4115	411.21
(B) Seedling survivorship^2^							
*Dodonaea*	+0.0001	−0.0104	+0.0218		−0.0199	+0.3987	1179.07
*Leptocophylla*	+0.0020		−0.0058	−0.1837			1398.52
*Morella*	−0.0005	−0.0198		−0.3337		−0.4267	1349.84

* Pluses and minuses denote the nature of the relationship between abundance/survivorship and the model effect; coefficients are shown if the relationship was significant at the *P* ≤ 0.05 level.

1 NA: Variables not measured, or not used, due to autocorrelation.

2 Seedling survivorship was not great enough in *Melinis* habitats for analysis.

### Outplant survival and growth

**Figure 3:**
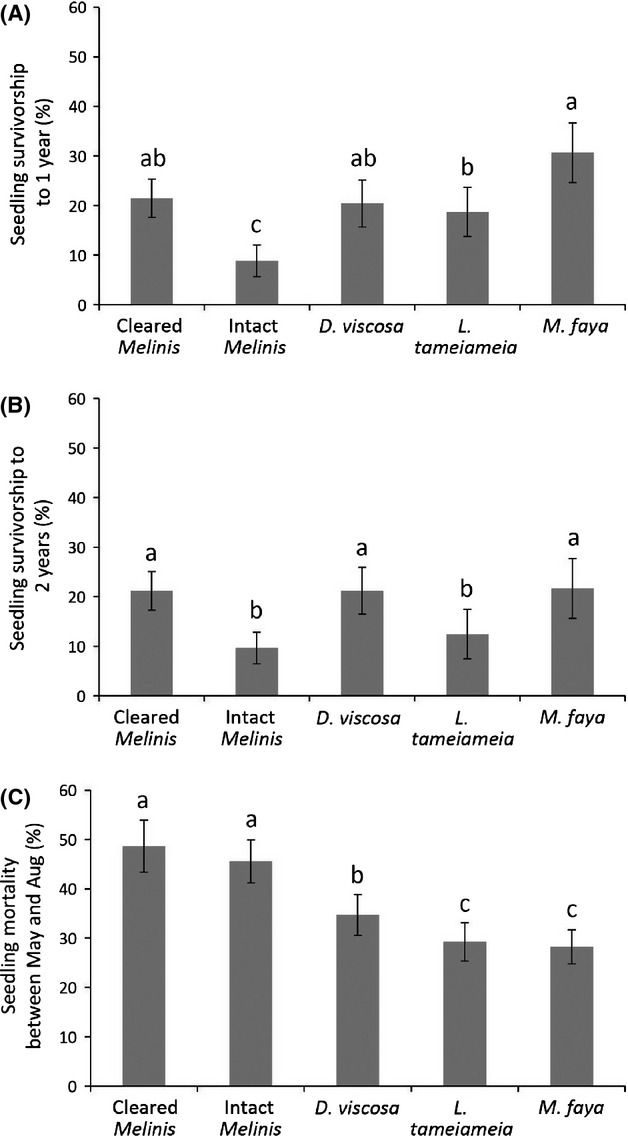
Seedling (A) survivorship after 1 year, (B) survivorship after 2 years, and (C) mortality between May and August, the harshest part of the growing year. Bars are means ± 1 SE. Letters represent significance at *P* ≤ 0.05, *n* = 7.

Survivorship of outplanted seedlings tended to be low (Fig.3). Across outplanted species and sites, we found the highest survivorship after 14 months was under *M. faya* (31%), and the lowest was within intact *Melinis* (9%) (Fig.3A). After 24 months, survival was equally high under *M. faya*, *D. viscosa*, and cleared *Melinis* (∼20%) (Fig.3B). During the hottest part of the first growing year (May–August 2012), nurse plants increased seedling survivorship compared to both cleared and intact *Melinis* (Fig.3C).

**Figure 4:**
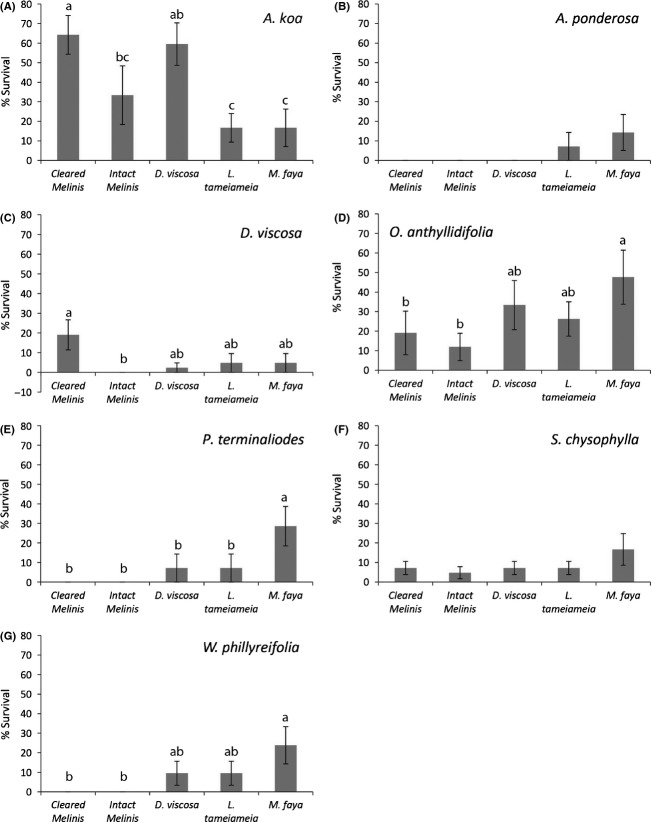
Survivorship after 2 years of seven species outplanted in different nurse plant (*Dodonaea viscosa, Leptocophylla tameiameia, and Morella faya*) and grass habitats (cleared *Melinis* and intact *Melinis*). Bars are means ± 1SE. Letters represent significance at *P* ≤ 0.05, *n* = 7.

Outplanted seedling species varied greatly in their response to the outplant habitats (Fig.4). Two species, *D. viscosa* and *A. koa*, showed the greatest outplant survivorship and growth in cleared plots, although both species showed some positive nurse plant responses. *D. viscosa* exhibited lower survival and growth rates in the intact *Melinis* matrix than under nurse species. *Acacia koa* had equally high survivorship and growth under *D. viscosa* as in cleared plots, but showed the lowest survivorship under *M. faya* and *L. tameiameia*, the two nurse shrubs that increased soil N (Table1). In contrast, *W. phillyreifolia*, *P. terminaliodes*, and *O. anthyllidifolia* only showed positive nurse plant effects. While all three species tended to survive the best under *M. faya*, the other nurse plant species also increased their survivorship relative to intact or cleared *Melinis*.

Outplanted species differed in which abiotic variables correlated with survivorship (Table3). Species responded differently to light: *A. koa, A. ponderosa*, and *D. viscosa* showed positive responses to high light, and *P. terminaliodes* and *W. phillyreifolia* showed negative responses. The latter two species tended to do better in cooler, moister, more shaded environments. Survivorship of most species correlated positively with soil C:N, a variable that covaried with soil % carbon (*R*^2^ = 0.37, *P* < 0.001), soil % nitrogen (*R*^2^ = 0.22, *P* < 0.001), and soil organic matter (*R*^2^ = 0.37, *P* < 0.001). This was due to *M. faya*, increasing %C, %N, and organic matter simultaneously under their canopies (Table1). Soil resin N was positively related to survivorship of *O. anthylidifolia*, *W. phillyraeifolia*, and *S. chrysophylla* (a presumed nitrogen fixer).

**Table 3:** Final glm models describing abiotic variables correlating with survivorship for seedling species in outplanting experiment

Seedling species	Potential Model Effects*					AIC
	Light (%)	Soil Temp (°C)	Soil moisture (%)	Soil C:N	Soil resin N (ppm)	
*Acacia*	+0.0098			+0.1474		1283.69
*Alphitonia*	−0.0984			−0.1346		417.55
*Dodonaea*	+0.0180			+0.1041		686.97
*Osteomeles*			+0.0673	+0.1014	+0.1486	945.26
*Pittosporum*	−0.0545	−0.2677	+0.0559	+0.4198		640.08
*Sophora*			+0.0979		+0.1475	559.85
*Wikstroemia*	−0.0353	−0.2533			+0.0946	545.51

* Pluses and minuses denote the nature of the relationship between abundance/survivorship and the model effect; coefficients are shown if the relationship was significant at the *P* ≤ 0.05 level.

Soil C:N and resin N were important for survival across all nurse species (Table4). In contrast, soil moisture only emerged as important under *L. tameiameia* and *M. faya*. Finally, light was negatively related to seedling survival under *M. faya*.

**Table 4:** Final glm models describing abiotic variables important to survivorship under different nurse shrub species in outplanting experiment

Nurse shrub species	Potential model effects*					AIC
	Light (%)	Soil Temp (°C)	Soil moisture (%)	Soil C:N	Soil resin N (ppm)	
*Dodonaea*				+0.2205	+0.5931	1843.48
*Leptocophylla*			+0.1682	+0.2255	+0.2677	1182.39
*Morella*		+0.04949	+0.0800	−0.4129	+0.2594	1598.84

* Plus and minus denote the nature of the relationship between abundance/survivorship and the model effect; coefficients are shown if the relationship was significant at the *P* ≤ 0.05 level.

### Greenhouse experiment

**Figure 5:**
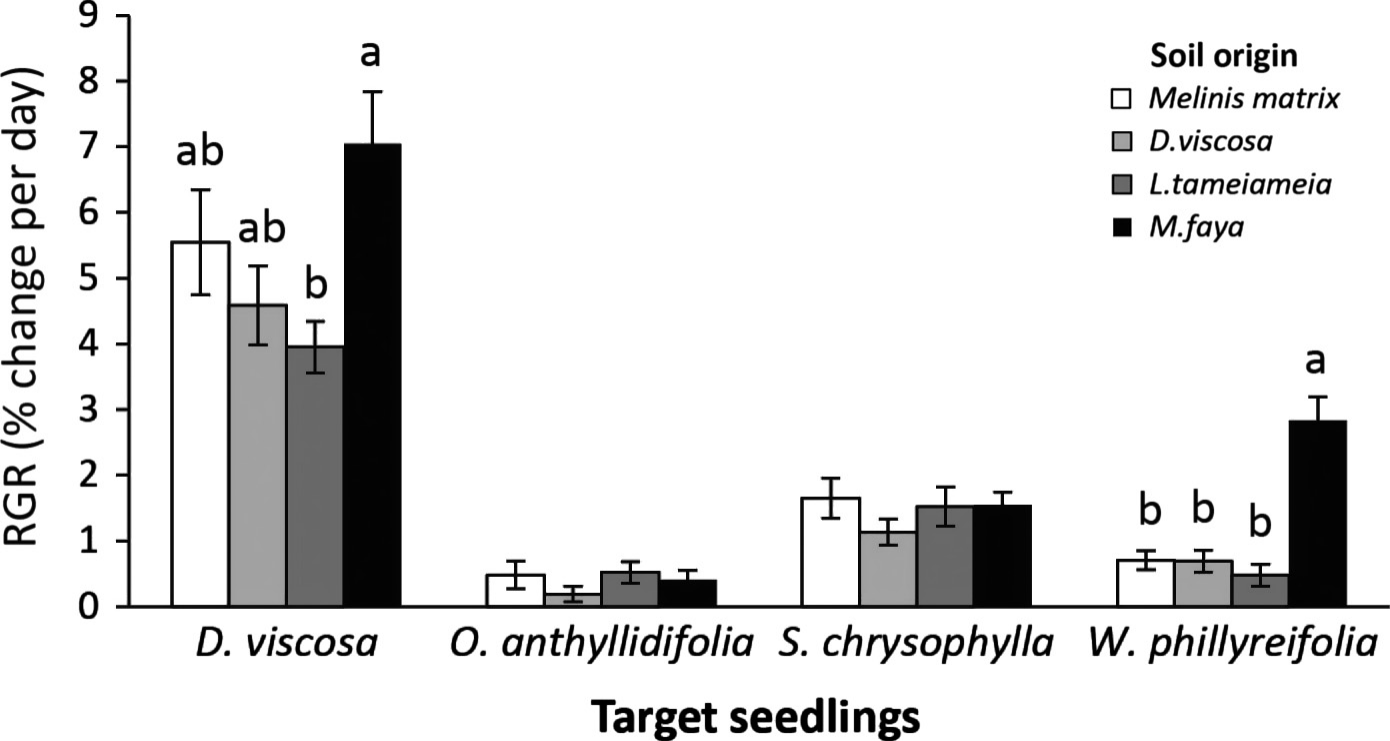
Relative growth rates of seedlings grown in a greenhouse experiment that used soil from different nurse plants and the exotic grass *Melinis*. Bars are means ± 1SE. One-way ANOVAs with soil as fixed effect: *Dodonaea viscosa*: *F*_3,32_ = 3.86, *P* = 0.02; *S. chrysophylla*: *F*_3,32_ = 0.89, *P* = 0.46; *Osteomeles anthyllidifolia*: *F*_3,32_ = 0.89, *P* = 0.46; *Wikstroemia phillyreifolia*: *F*_3,32_ = 22.31, *P* < 0.001. Letters represent differences at *P* ≥ 0.05 for post hoc Tukey tests.

Two of the four species tested, *D. viscosa* and *W. phillyreifolia*, showed higher relative growth rates in *M. faya* soil than the other soils, but no other soil effects were evident (Fig.5). *Myrica faya* soil had the highest levels of resin-captured nitrogen (1068 ± 61 μg/mL vs. 741 ± 18, 718 ± 10, and 691 ± 10 μg/mL for *D. viscosa*, *L. tameiameia*, and *Melinis* matrix, respectively, 1-way ANOVA: df = 3,13, *P* < 0.001). Growth rates of *S. chrysophylla* (*P* = 0.457) and *O. anthyllidifolia* (*P* = 0.457) seedlings did not differ between soil types (Fig.5), a surprising result given that these species tended to have higher survivorship in soils with greater resin N (Table3).

## Discussion

We found that nurse plants may be a useful tool for enhancing plant establishment, but their role depended on the outplanted species. Plant responses to nurse environments varied across species and growth stage, yet certain patterns emerged that may guide management efforts. If these degraded sites are left alone, natural regeneration will be limited because dispersal of all species except *D. viscosa* is poor, suggesting that spread of native shrubs from adult foci will be slow. Planting under nurse shrubs was not beneficial for early successional species, such as *D. viscosa*, which survived better in open microsites. Otherwise, most species, excepting *S. *c*hrysophylla* and *A. ponderosa*, benefited from nurse plants to some degree. *Acacia koa*, an N-fixing legume, benefited from *D. viscosa* nurse plants, but showed poor survivorship under *M. faya* and *L. tameiameia*, the nurse species with elevated soil N. Overall, the worst habitat for seedling survival was the existing *Melinis* matrix; thus, any strategy that lessens exotic grass dominance would benefit restoration of native species.

### Natural regeneration

Existing adult native shrubs act as both seed sources and nurse habitats increasing shrub seedling abundance and survival relative to the *Melinis* matrix. However, our data suggest that active restoration is needed to achieve a diverse community structure because most shrub species recruited beneath adults of the same species and no species other than *D. viscosa*, *L. tameiameia*, and *M. faya* were found beneath the monitored canopies. This suggests that dispersal limitation and competition from grasses are hindering native shrub recruitment. Indeed, *Melinis* habitats ranked poorly for abundance and survival of natural seedlings, corroborating that grass competition plays a large role in preventing native shrub re-establishment (Hughes and Vitousek 1993; Yelenik and D'Antonio 2013).

Our data suggest that without active restoration, the only native shrub to colonize the *Melinis* matrix will be *D. viscosa*. Other work has shown that *D. viscosa* has substantially increased in percent cover since the 1990s in these sites (D'Antonio et al. 2011; Yelenik and D'Antonio 2013). This is partly due to its winged seed pods, and also due to the fact that *Melinis* abundance has declined over time as resources have changed (Yelenik and D'Antonio 2013), leading to patches of reduced grass cover and reduced competition. In contrast, many of the other species produce berries that fall directly underneath the canopy and are potentially eaten by non-native rodents (Anderson 2000; Pratt et al. 2011). While native frugivorous birds may have once dispersed seed, they are locally extirpated, leading to recruitment limitation of native species in lowland Hawaiian dry forest (Vieira and Scariot 2006; Chimera and Drake 2011). Besides *D. viscosa*, only *L. tameiameia* is still found in these sites, while the other outplanted species have been lost from the local species pool due to fire or other factors. Therefore, outplanting or manual seeding is necessary to create a more diverse shrub community.

Abiotic factors that correlate with natural recruitment can depend on the life stage examined. For example, shrub size was negatively associated with seedling numbers under *D. viscosa*, but positively associated with seedling survival (Table2A, B). If outplanting is used for restoration, then planting under large shrubs that decrease light and ameliorate harsh conditions may be the most beneficial approach. This may help to decrease water stress in the outplanted species, some of which have been shown to have increased survival and growth with watering in Hawaiian dry forest restoration studies (Cabin et al. 2002a). Alternatively, if direct seeding is used to increase diversity and abundance, then seeding under small nurse shrubs with moderate light transmittance may be the most beneficial. In a meta-analysis, Gomez-Aparicio (2009) saw shifts between competition and facilitation across seedling life stages, which is consistent with our observations.

*Morella faya*, a well-studied (Vitousek and Walker 1989) exotic N-fixing tree, is rapidly invading these sites (Asner et al. 2010; Yelenik and D'Antonio 2013). Yet, we found 99% of the *M. faya* seedlings under itself (Fig.1B), and only 3.5% of these survived. High mortality is probably due to self-shading and the robust swards of *Melinis* that develop around *M. faya* trees (Adler et al. 1998; Yelenik and D'Antonio 2013). *Morella faya*, a bird-dispersed species, invades from under live and dead individuals of *M. polymorpha*, the former native canopy dominant (Smathers and Gardner 1978; Vitousek and Walker 1989) where we did not census seedlings. Therefore, while *D. viscosa* is the only native shrub that is actively colonizing our sites, *M. faya* invasion should not be overlooked when considering the long-term fate of the ecosystem.

### Nurse plant effects

Understanding when nurse plants are useful for restoration hinges on understanding how traits of target seedlings and nurse species match (Gomez-Aparicio 2009). Our results fit with the work of others in showing that the successional association of outplanted species is crucial for predicting whether nurse plants increase seedling survivorship (Gomez-Aparicio et al. 2004; Yang et al. 2010). *Dodonaea viscosa*, a species that is an early colonizer of open lava flows and postfire environments had the highest survivorship and growth in open microsites despite the fact that in the greenhouse, it responded positively to *M. faya* soil (Fig. S1, Table3). This latter effect was likely due to higher soil nitrogen given that it responds positively to fertilization in the field (Yelenik and D'Antonio 2013). Therefore, while nurse shrubs may foster soils that are facilitative, the net outcome of the interaction due to light, litter, etc. may still be negative as has been shown for other early successional species (Callaway and Walker 1997; Padilla and Pugnaire 2006).

Species that did not respond positively to light—presumably later successional ones—tended to have higher survivorship as a function of soil moisture and nitrogen (Table3). Indeed, target seedling species considered as later successional (*P. terminaliodes*, *O. anthyllidifolia*, and *W. phillyreifolia*) survived best under nurse plants. These three species fared better under *M. faya* than under *L. tameiaeia* or *D. viscosa*, and there was substantial evidence that this result was due to high nitrogen, and perhaps shade: *Wikstroemia* had higher growth rates in the greenhouse with *M. faya* soil, *O. anthyllidifolia* responded positively to nitrogen fertilizer (Yelenik and D'Antonio 2013) and showed a positive relationship between growth and soil resin N (Table3), and survivorship of *P. terminaliodes* related positively to soil C:N and moisture (Table3). Similarly, in southwest Spain, Gomez-Aparicio et al. (2004) showed that N-fixing nurse plants were the most beneficial for target seedlings.

In contrast, outplants of *A. koa*, an N-fixing tree, had the lowest survivorship and growth under *L. tameiameia* and *M. faya*, nurse species that increased soil N (Table1). In a separate study, *A. koa* growth rates were lower in N-fertilized compared to unfertilized plots because of increased *Melinis* growth (Yelenik and D'Antonio 2013). Here, *Melinis* was cleared before outplanting, so the lower survival and growth of *A. koa* under high N shrubs may be due to other reasons such as inhibition of N fixation by soil N (Vitousek et al. 2002). In addition, N-fixing trees tend to be early successional and shade intolerant, particularly in temperate ecosystems (Menge et al. 2010), and thus may be harmed by low light under nurse species. *Sophora chyrsophylla*, however, which is also a legume and nodulates in the greenhouse (D'Antonio unpublished data), showed no response to nurse plants. Because this tree has low growth and survival compared to *A. koa* (Fig. S1), and seedlings respond positively to N fertilizer (Yelenik and D'Antonio, unpublished data), using family (e.g. Fabaceae) to match a species to a nurse environment should be done with caution.

The *Melinis* matrix was the worst habitat for outplant survivorship (Fig.4). Both cleared and intact *Melinis* were associated with high seedling mortality during the summer months. It is probable that outplanted seedlings experienced high water stress in these environments due to either high light (cleared) or high competition (intact). Grasses are known to have dense fibrous roots in the upper soil profile, making them successful competitors against woody-plant seedlings (Scholes and Archer 1997). A meta-analysis of nurse plant effects across many ecosystems showed that herbaceous neighbors tended to decrease outplant survival, and grasses showed the largest of these negative effects, whereas shrub neighbors tended to increase outplant growth and survival (Gomez-Aparicio 2009).

Nurse plants offer a benefit for shade-tolerant species, but these effects were not as clear as we hypothesized. Nurse plants form a complex study system because they combine positive and negative interactions that are often inter-related, yet it is the net effect that matters (Harper 1977; Callaway and Walker 1997; Padilla and Pugnaire 2006; Brooker et al. 2007). For example, reduced light from nurse plant shading decreases evaporation of water from soil, but simultaneously reduces photosynthesis. In addition, the effects of abiotic variables are often nonlinear and therefore can create complex outcomes. Soil litter layers created by nurse species, for example, are increasingly helpful in buffering soil temperature and water loss to a point, after which they physically cover seedlings, having a negative impact. Finally, abiotic variables may have differing effects depending on the life stage of the target seedling (Miriti 2006).

### Management Implications

Because *D. viscosa* and *M. faya* are the only species actively increasing in abundance in the absence of restoration (D'Antonio et al. 2011; Yelenik and D'Antonio 2013), it is of particular interest to evaluate these as nurse plants for future projects (Smit and Ruifrok 2011). Although *M. faya* had the highest survivorship of outplantings after 1 year, after 2 years, survivorship was equal to the other nurse treatments (Fig.3). This could have to do with abiotic effects shifting in direction and importance over the life of the seedling (Table2). *M. faya* size was positively related to natural seedling abundance, but negatively related to natural seedling survival, consistent with high N, low temperature microclimates being a net benefit at the earliest life stage, but not for survival. It should also be noted that our seedlings in the outplant study were planted at the edge of the *M. faya* canopy, which may initially confer enough light for growth with some protective shading, but *M. faya* canopies quickly widen (Yelenik and D'Antonio 2013) decreasing light to detrimental levels. Observationally, older shrubs growing under *M. faya* show clear signs of light limitation (pers. observations all authors, SY, ND, and CD).

Thus, although late-successional outplanted species showed better survivorship under *M. faya* than under *D. viscosa* (Fig.3), we strongly caution that using *M. faya* as a nurse plant may not be successful in the long term. *Morella faya* is considered a highly invasive species and is spreading across Hawai'i Volcanoes National Park and surrounding areas at a fast pace (Loh and Daehler 2008). For example, our sites have shown an increase in *M. faya* biomass along long-term transects from 0 g/m^2^ in 1995 to 2000 g/m^2^ in 2011 (D'Antonio et al. 2011; Yelenik and D'Antonio 2013). Instead, a potential strategy may be to plant under existing *M. faya* canopies, while girdling the trees and leaving them standing to provide shade. At nearby wetter sites, girdling *M. faya* was found to benefit existing native species in the understory, but at our sites because of the lack of a native seedbank, girdling alone would likely facilitate herbaceous weeds including *Melinis* (Adler et al. 1998; Loh and Daehler 2008). Thus, coupling of outplanting with girdling may be a more general strategy for managing N-fixing invasive woody species.

*Dodonaea viscosa* also showed potential as a nurse species and is currently being used as a nurse shrub in other parts of Hawai'i Volcanoes National Park (McDaniel, pers. com), and in a dry forest site on Maui (Medeiros et al. 2014). On Maui, the dominant exotic rhizomatous grass (*Cenchrus clandestinus*) was killed with herbicide, and dense *D. viscosa* established. Importantly, 12 species of native tree/shrub increased in abundance and/or dispersed into the site without augmentation over 15 years, and other rare species have successfully been outplanted (Medeiros et al. 2014). While this site differs in climate, the study results suggest that augmenting *D. viscosa* abundance by reducing grass cover and seeding will eventually help other native shrubs colonize. Higher canopy cover of *D. viscosa* will help to decrease light and increase soil C:N, organic matter, and moisture retention, as well as create competitive environments for exotic grasses and shrubs, such as *Melinis* and *M. faya*. In addition, it is easy to obtain seed, propagate, and grow *D. viscosa* in the greenhouse (Lilleeng-Rosenberger 2005; Medeiros et al. 2014), making it an accessible and cost-effective restoration option.

In contrast, existing *L. tameiameia* shrubs may be effective nurse plants, but they are difficult to propagate and they grow slowly. Indeed, we had limited success growing this species from seed and instead collected seedlings from under parent shrubs in the field and used these for outplanting in a separate study (Yelenik and D'Antonio 2013). In addition, seedlings obtained very little biomass during the study. Thus, the idea of outplanting *L. tameiameia* en masse to act as nurse plants for the future may not be very viable. However, existing shrubs on the landscape should be used as nurse plants during outplanting efforts, although these tend to be sparse in sites that have burned and been invaded by *Melinis*.

## Conclusions

Our work and that of others (e.g., Miriti 2006; Aerts et al. 2007; Gomez-Aparicio 2009; Medeiros et al. 2014) suggests that if used properly, nurse plants may be an effective restoration tool. Understanding the successional or shade-tolerant nature of target outplant species is important when considering the benefit of nurse plants. The use of nurse plants should be strategic and based on matching of outplant species to nurse environments. It should also be kept in mind that net effects of nurse plants may also shift across environmental gradients (Maestre et al. 2009), which is especially important in Hawai'i due to steep precipitation and temperature gradients (Giambelluca et al. 2013). The relationship of nurse plant benefits to environmental harshness has yet to be tested in the Hawaiian Islands despite the prevalence of degraded, grass invaded habitat.

## Supporting Information

**Figure S1.** Relative growth rates of outplant seedlings after 5 months of growth.

**Table S1.** Table of seedling outplant species with their relevant characteristics. **Table S2**. Seed treatment protocols and average seedling heights.
