# Supplementary figures and images for: Evaluating nurse plants for restoring native woody species to degraded subtropical woodlands
Source: Ecol Evol. 2014 Dec 23;5(2):300–13. doi: 10.1002/ece3.1294 (PMC4314263; doi:10.1002/ece3.1294)

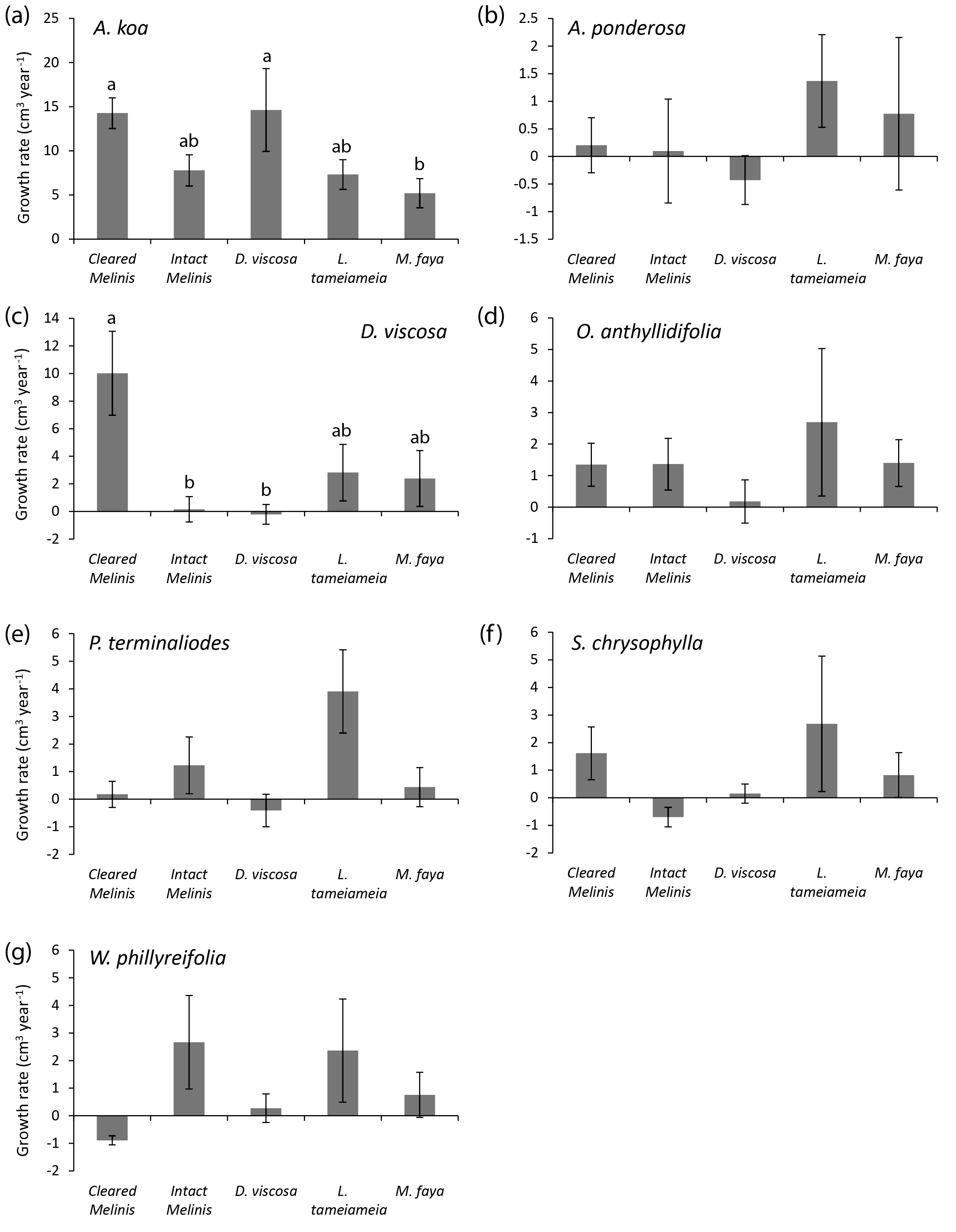

Supplement: Supplementary file 1 [file ece30005-0300-sd1.tif]
