# Supplementary material for: Evaluating nurse plants for restoring native woody species to degraded subtropical woodlands
Source: Ecol Evol. 2014 Dec 23;5(2):300–13. doi: 10.1002/ece3.1294 (PMC4314263; doi:10.1002/ece3.1294)
Supplement: Supplementary file 2 [file ece30005-0300-sd2.doc]

**Table S1**: Table of seedling out plant species with their relevant characteristics

| **Seedling species** | **Family** | **Common name** | **Notable characteristics** |
| --- | --- | --- | --- |
| *Acacia koa* | Fabaceae | koa | N-fixing tree, relatively fast growing, found close to sites but not naturally recruiting there, common restoration species |
| *Alphitonia ponderosa* | Rhamnaceae | kauila | Tree, dry to mesic forests, not currently found in the intact woodland near our sites, threatened species |
| *Dodonaea viscosa* | Sapindaceae | ʻaʻaliʻi | Common, early successional shrub, winged seed, found in nearby burned and unburned woodland |
| *Osteomeles anthyllidifolia* | Rosaceae | ʻūlei | Common shrub in unburned woodland, sprawling growth form, seed in small fruit |
| *Pittosporum terminaliodes* | Pittosporaceae | hōʻawa | Tree, dry to mesic forests, found in nearby unburned woodland, large seeded, threatened species |
| *Sophora chrysophylla* | Fabaceae | māmane | N-fixing tree, relatively slow growing, found in nearby unburned woodland |
| *Wikstroemia phillyreifolia* | Thymelaeaceae | ʻākia | Understory shrub in unburned woodland, seed in small fruit |

**Table S2**: Seed treatment protocols and average seedling heights in outplanting study*

| **Species** | **Treatment regime** | **Average seedling height (cm)** |
| --- | --- | --- |
| *Acacia koa* | Removed from pods and soaked in water for 24 hours. Non-viable seeds were discarded†. Viable seeds were sown ¼ in. below the media’s surface. | 7 |
| *Alphitonia ponderosa* | Removed from their hard fruits (using a mallet). Soaked in water for 24 hours. Seed ends were clipped. Sown ½ in. below the media's surface. | 2.5 |
| *Dodonaea viscosa* | Removed from their capsules and soaked in water for 24 hours. Non-viable seeds were discarded. Viable seeds surface sown. | 2.2 |
| *Osteomeles anthyllidifolia* | Removed from their fruit and soaked in water for 24 hours. Non-viable seeds were discarded†. Viable seeds were surface sown. | 2.7 |
| *Pittosporum terminaliodes* | Removed from their capsules, rinsed with soap/water solution, and soaked in water for 24 hours. Sown ½ in. below the media's surface. | 2.6 |
| *Sophora chrysophylla* | Removed from pods. Acid scarified using a sulfuric acid treatment. Surface sown. | 4.3 |
| *Wikstroemia phyllraeifolia* | Removed from their fruits and soaked in water for 24 hours. Non-viable seeds were discarded†. Viable seeds surface sown. | 2.2 |

* Modified from Lileeng-Rosenberger (2005)

† Determination of non-viability was made when seeds would float to the water's surface
